# Framework for evaluating external and internal parameters associated with Sea Based Container Culture (SBCC): Towards understanding rearing success in European lobsters (*Homarus gammarus*)

**DOI:** 10.1016/j.aquaeng.2018.09.005

**Published:** 2018-11

**Authors:** Peter Halswell, Carly L. Daniels, Lars Johanning

**Affiliations:** a College of Engineering, Mathematics and Physical Sciences, University of Exeter, Exeter, UK; b National Lobster Hatchery, Padstow, Cornwall, UK

**Keywords:** ADCP, acoustic doppler current profiler, DOD, issolved oxygen, EVP, external velocity profile, FaBTest, falmouth bay test site, IVP, internal velocity profile, RAS, recirculating aquaculture systems, RMS, root mean squared, SBCC, sea based container culture, SWMTF, south west mooring test facility, European lobster, On-growing, Mariculture, Hydrodynamic, Rearing success

## Abstract

Sea Based Container Culture (SBCC) is a mariculture technique that relies on the natural maintenance of environmental conditions, such as Dissolved Oxygen (DO) concentration and feed availability. This paper discusses a framework to evaluate the rearing success of European Lobsters (*Homarus gammarus*) in SBCC based on temporal and spatial variations of external parameters, including current velocity, wave velocity, turbulent fluctuations and dissolved oxygen concentrations. The temporal variations considered annual changes to the environment and the effect of biofouling growth, and the spatial variations considered the geographical location (case study of Falmouth bay, Cornwall) and vertical position in the water column. The internal parameters of the containers were modelled using transfer functions derived from previous experimental data. The internal parameters were compared to rearing limitations selected from available literature, which included foraging and mobility behaviours, and DO consumption. The time that internal parameters exceeded the rearing limitations was quantified, allowing rearing success to be predicted.

This paper uses a case study of external parameters measured in Cornish waters, UK, to demonstrate the framework methodology. The framework showed that in situ measurements of current, wave and turbulence could be used to predict the internal parameters of SBCC containers, which can be used to predict theoretical rearing success based on rearing limitations. The framework indicated that DO concentrations within the containers should not affect rearing success; however, the foraging and mobility limits were exceeded by 0 to 30% of the time (depending on vertical position in the water column and assessment method). The paper aims to demonstrate the generic framework methodology and understands its limitations in predicting rearing success. The framework provides a tool to optimise the SBCC design for spatial and temporal varying conditions related to a geographical location or (vice versa) identify suitable mariculture sites based on SBCC design and environmental conditions. Additionally, the framework can optimise the vertical position of the SBCC in the water column and identify, from parameters considered, those that are most likely to affect rearing success.

## Highlights

• A novel framework for evaluating the effect of external parameters on rearing success of European lobsters in SBCC systems has been presented.
• Current, wave and turbulent measurements from Cornish waters were used to define external parameters, and internal parameters were predicted from external parameters.
• Rearing limitations of European lobsters were defined from available literature based on DO consumption and, foraging and mobility behaviours.
• The framework assessed and optimised the geographical location of SBCC systems, the vertical position in the water column and the SBCC design.

## 1 Statement of relevance

This study demonstrates the use of mathematical predictive tools to model the success of novel SBCC systems, utilising a case study in Cornish waters, UK, to predict the effect of external parameters on lobster rearing success.

## 2 Introduction

The world population is forecast to increase by 2.3 billion people by 2050 to 9.6 billion (DESA, U.N., 2013) putting increasing pressure on existing protein sources. With terrestrial resources such as agricultural land being limited and many natural aquatic resources already over-exploited, it is becoming more apparent that aquaculture could provide a sustainable, secure food source to help alleviate these growing pressures. The upward trend in aquaculture production is already underway with global captured fisheries production increasing by only 0.4% between 2006 and 2011 compared to the growth in aquaculture of 34.5% over the same period (Mathiesen, 2012). The European lobster (*Homarus gammarus*) shows potential as a candidate for novel mariculture, termed Sea Based Container Culture (SBCC), over other unexploited species due to its high global prices (Drengstig and Bergheim, 2013). Capture fisheries supply of European Lobster is currently in the region of 5000 t per annum, originating mainly from the UK and Ireland (http://www.fao.org/fishery/species/2648/en).

The current method of rearing clawed lobsters utilises land based Recirculating Aquaculture Systems (RAS) mainly for stock enhancement purposes where lobsters are reared to early juvenile stages and released into the wild to supplement natural stocks. Work in Norway has demonstrated the feasibility of rearing lobsters to market size (Drengstig and Bergheim, 2013), however, biological and technological barriers as well as excessive capital investment has to date deterred further progression of RAS for lobster culture. A key advantage of SBCC over RAS relates to natural maintenance of the environmental conditions in terms of water quality (temperature and salinity), Dissolved Oxygen (DO) availability, feed availability, sediment removal and excrement removal (Uglem et al., 2006; Perez-Benavente et al., 2010; Browne et al., 2011; Daniels et al., 2015). Stress in aquatic organisms occurs when external factors result in physiological processes being extended beyond the normal range of tolerance (Iwama, 1997), which can have adverse effects on metabolism with the potential to significantly affect rearing success (Calabrese et al., 1977; Pickering, 1993; Petes et al., 2007). DO has been highlighted as a critical external factor in lobster culture (Bignell et al., 2016). Flow delivers oxygenated water and feed whilst also disposing of sediment, deoxygenated water and waste products (Drengstig and Bergheim, 2013; Uglem et al., 2006; Burton, 2003). Recommended flow rates for rearing lobster ranges from 4 L min^−1^ (Beal et al., 2002) to 100 L min^−1^ (Drengstig and Bergheim, 2013), though this will vary according to the biological load in any given environment. However, flow velocity affects mobility and behaviour of lobsters but can also cause physical exhaustion, damage and/or fatalities (Hamelo, 2006; Galparsoro et al., 2009; Howard and Nunny, 1983; Smith et al., 1999). Flow velocity exceeding 0.27 m/s can severely impair a lobsters use of their olfactory appendages (Howard and Nunny, 1983), those appendages vital for actively sampling odor-bearing fluid from the environments to locate food, identify mates and find suitable habitats (Reidenbach et al., 2008). Howard and Nunny also showed that mobility was severely impaired at higher velocities but the feeding behaviour, specifically foraging, increases when the flow velocity reduced below 0.1 m/s (Howard and Nunny, 1983).

The rearing success in SBCC systems is not fully understood, but it will vary depending on an array of interconnecting external environmental parameters, which in turn naturally maintain the internal rearing environment experienced by the lobster. This paper therefore aims to provide the first framework to evaluate the effect of external environmental parameters on the rearing success in SBCC systems; focusing on the connection between hydrodynamics, DO and behaviour. The authors acknowledge the existence of alterative and additional external parameters (e.g. food availability, food type and food quality) and as such the framework will be developed with flexibility, so it can be expanded to include further parameters as information becomes available. The framework is aimed at all stakeholders involved in mariculture and SBCC from farmers, researchers or stock enhancers, with focus on European lobsters. The framework will be demonstrated using a case study of Falmouth bay, a potential deployment site for SBCC farms in Cornwall, UK.

## 3 Material and methods

### 3.1 Framework

**Fig. 1:**
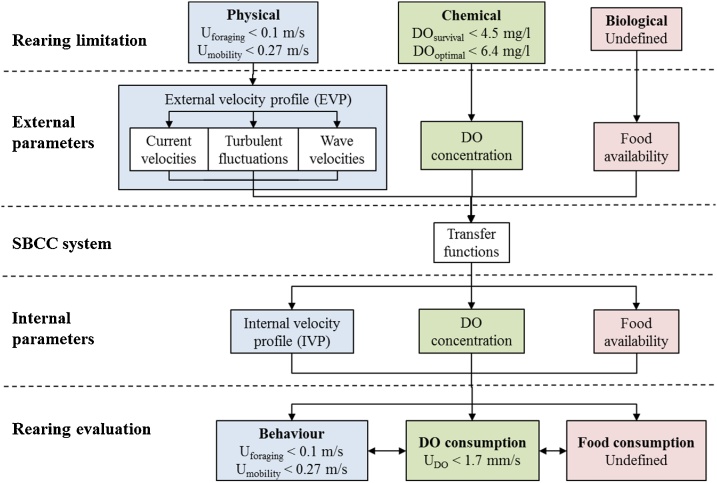
Framework developed to evaluate the rearing success in SBCC systems. The framework analytically predicts internal parameters from external environmental parameters using transfer functions and consequently infers rearing success. Where U is a flow velocity limit and DO is a dissolved oxygen concentration limit.

The evaluation framework (Fig. 1) to predict the rearing success of European lobsters in SBCC systems had five main steps; rearing limitations, external parameters, SBCC system, internal parameters and rearing evaluation. The framework starts by selecting the rearing limitations suitable for inclusion, separated into physical, biological and chemical categories; the categories allow for additional parameters to be added once knowledge gaps are filled. Suitable rearing limitations are quantifiable parameters that affect the rearing, growth or survival of lobsters. The limitation should be scientifically proven and must be comparable to an external parameter. Flow velocity limitations (U) (Section 2.2.1) and DO concentration (Section 2.2.2) were chosen as suitable rearing limitations for this case study.

Next the spatial and temporal variations of external parameters relating to the rearing limitations were defined. The physical external parameters evaluated in the present study were hydrodynamic, namely current velocity, wave velocity and turbulent fluctuation, the combination of which was termed External Velocity Profile (EVP). The remaining external parameter considered was DO concentration (chemical). As common in many marine taxa, food sources for lobsters vary throughout their life cycle and, though no quantitative assessment of feed availability has been undertaken for lobster SBCC systems, food has been shown to have spatial and temporal variations (Daniels et al., 2015). Additionally, the DO concentration followed an annual variation (Fig. 6) and the concentration will vary through the water column. Therefore, all external parameters possess spatial and temporal variations and these were used to achieve the greatest understanding and accuracy. The spatial definition must cover deployment area to be evaluated and define the depth-dependent variation, whilst the temporal definition must cover at least 1 year to included yearly variation.

Transfer functions (mathematical method of relating an output to an input) were used to convert external parameters into internal parameters. The transfer functions for the velocity profiles were derived from a previous study (Halswell et al., 2016), based on the porosity and body shape of SBCC containers; here linear transfer functions were used. Biofouling growth on SBCC containers causes a temporal variation of transfer functions, which was accounted for using separate transfer functions for percentage of biofouling coverage. Transfer functions were also used to convert parameter units allowing internal parameters to be cross-evaluated. This will be demonstrated in Section 2.4.2, where DO concentration is converted to flow velocity based on the rate of DO consumption.

The internal parameters were then calculated from the external parameters. The Internal Velocity Profile (IVP)^1^ was theoretically predicted from the EVP and the DO concentration limits were converted to velocity limits for evaluation.

Finally, the framework compared the IVP to the rearing limitations, which have all been converted to velocity limits, to predict the theoretical rearing success in the SBCC system. The framework was used to consider the rearing success in terms of geographical location, vertical position in the water column and point in time (i.e. extreme yearly conditions and biofouling growth). Statistical methods (percentage of time and longest continuous period of time) were used to quantify the time that internal parameters are above or below the behaviour or DO velocity limits allowing rearing success to be theoretically quantified.

### 3.2 Rearing limitations

#### 3.2.1 Behaviour

A current flume experiment involving live adult lobsters demonstrated behavioural responses to varying flow velocities on two seabed substrates (Howard and Nunny, 1983). Flow velocity exceeding 0.27 m/s can severely impair mobility and olfactory senses, termed mobility velocity limit. Additionally, foraging increases when the flow velocity reduced below 0.1 m/s, named foraging velocity limit. The turbulent fluctuations of the current flume used by Howard and Nunny were not quantified and the effect of turbulence on behaviour was not discussed; thus the effect of turbulence on behaviour will not be explored in this paper. This framework considers post larval lobsters, and as such the authors accept some limits to using data associated with adult lobsters, though without further investigation; this is the most relevant data available for the case study examined here.

#### 3.2.2 Dissolved oxygen consumption

Previous studies considering the effects of DO concentration on the growth and survival of lobsters have stated that the DO concentration at 10 °C must be greater than 4.5 mg/l (Beard and McGregor, 2004) for survival and 6.4 mg/l for optimal growth (Drengstig and Bergheim, 2013). This provided two limits for rearing success in SBCC systems based on DO concentrations. Fresh, oxygenated water is supplied at a rate derived from the IVP (driven by the EVP), thus the supply rate of oxygenated water must be greater than the consumption rate of oxygen. It must be considered that temperature affects the available DO concentration in seawater (Beard and McGregor, 2004), as such these limits present limitations in their applicability to all situations. Complications are also presented by factors effecting oxygen consumption, such as organism size and digestive state as well as biofouling in container systems; these are not addressed in the current model.

### 3.3 External parameters

**Fig. 2:**
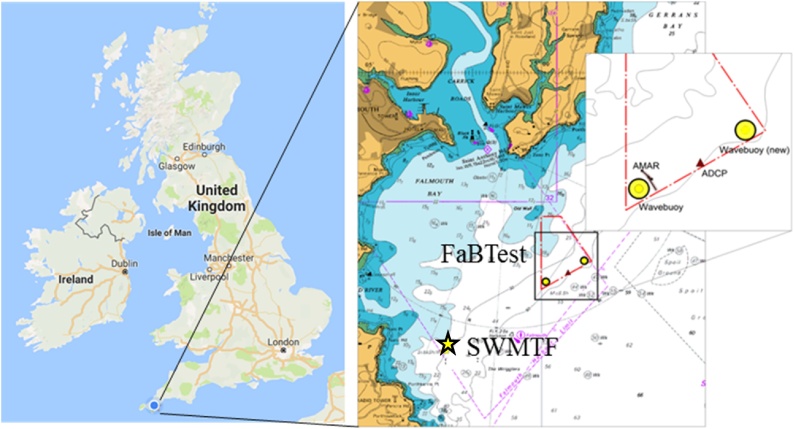
Locations of FaBTest and SWMTF field sites in Falmouth Bay, UK.

Current velocities, wave velocities, turbulence fluctuations and DO concentrations have been collected for various field sites to provide a case study for Cornwall, UK. The case study presented here is based on long term current and wave measurements from two demonstration sites in Falmouth bay (Fig. 2), Cornwall, UK: the Falmouth Bay Test site (FaBTest) and South West Mooring Test Facility (SWMTF). Turbulence data was measured at the Wave Hub site, located 16 km northwest of St. Ives, Cornwall, UK. Oceanic DO concentration was sampled in Falmouth Bay (Data obtained from public sector information licensed under the Open Government Licence v3.0).

#### 3.3.1 Current velocity

Current velocities were measured with an Acoustic Doppler Current Profiler (ADCP) that provided profiles of current speed and direction through the water column. The ADCP used was a 600 kHz Workhorse Sentinel (2009) manufactured by Teledyne Technologies Incorporated, CA, USA, with a stated accuracy of ±(0.03 m/s + 0.3% of measurement value), (Teledyne and Instruments, 2009). Bin height (sample height) was 0.5 m and first bin was 1.24 m above the sea bed. Sample frequency was 2 Hz and data was averaged over a 10 min period when calculating mean flow parameters. Current velocities were recorded at FaBTest between 25/07/2011 and 08/08/2011 and at SWMTF between 16/09/2010 and 03/06/2011 to characterise the current profile at each site.

**Fig. 3:**
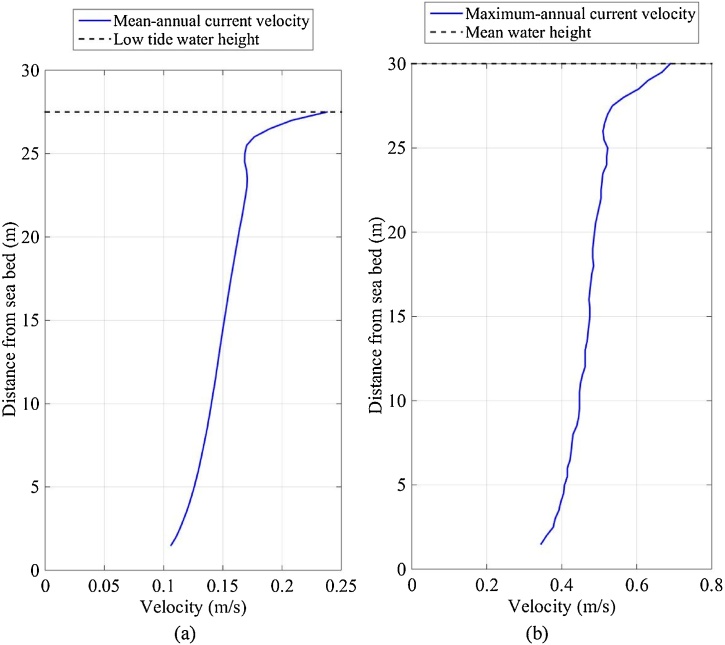
EVP of (a) the mean-annual current magnitude measured between 16/09/2010 and 03/06/2011, and (b) the maximum-annual current magnitude measured on 10/03/2013 between 19:36 and 21:16 at SWMTF.

The depth-averaged current velocities measured at FaBTest showed semidiurnal patterns (caused by the earth’s rotation) and a half-monthly pattern of spring and neap tides (caused by the moon’s orbital period). Current velocities varied vertically through the water column due to factors such as wind, waves, boundary layers and bathymetry; demonstrated by the vertical-profile of the mean-annual current magnitude measured at SWMTF (Fig. 3a). A maximum velocity of 0.24 m/s is shown at the sea surface and a minimum of 0.11 m/s near the sea bed (Fig. 3a). The small velocity dip (25 m from the sea bed) was most likely caused by the prevailing south-westerly winds in Falmouth bay (van Nieuwkoop et al., 2013). The mean-annual current profile does not consider daily and monthly tidal patterns or extreme wind and waves. The maximum-annual current profile (Fig. 3b) shows a vertical variation of velocity, a maximum velocity of 0.69 m/s at the water surface and a minimum of velocity of 0.35 m/s near the seabed. The maximum-annual current profile was captured on 10/03/2013 between 19:36 and 21:16 during a spring tide and extreme waves (significant wave height was 3.99 m, maximum wave height was 6.09 m, peak wave period was 8.1 s and mean wave period was 7.4 s).

#### 3.3.2 Wave velocity

Wave parameters were collected using a directional Seawatch mini II wave buoy manufactured by Fugro OCEANOR AS, Norway, at the FaBTest site from March 2012 to publication date, providing a wide range of measured conditions including significant storms during the winter 2013/2014 (Sanmuganathan, 2009). Wave parameters, such as wave height (*H*) and wave period (*T*), were calculate from the first 17.07 min of every half hour (2048 readings). The summary parameters were processed and analysed by an algorithm developed by the Offshore Renewable Energy group at the University of Exeter (Ashton, 2011).

Wave velocity profiles have been analytically predicted (Sarpkaya and Isaacson, 1981; page 158) and the method depends on the ratio of water depth to wave length. Deep water waves occur if the water depth is greater than half the wave length. Deep water waves create circular orbital velocities that have equal horizontal (u) and vertical (w) velocities (Eqs. (1) and (2)); whilst shallow water waves generate elliptic orbital velocities with larger horizontal velocity than the vertical velocity.

(1) $${\text{u}}_{\text{deep}}=\frac{\text{πH}}{\text{T}}{\text{e}}^{\text{kz}}\text{cosθ}$$

(2) $${\text{w}}_{\text{deep}}\text{=}\frac{\text{πH}}{\text{T}}{\text{e}}^{\text{kz}}\text{sinθ}$$

Where, *u* is horizontal and *w* is vertical particle velocity (m/s), H is wave height (m), T is the wave period (s), k is wave number and θ is phase angle (°). The water depth at a given time is *d* (m) and *z* is a specific vertical position in the water column (m). For a simplified approach k can be expressed through the dispersion relation (Eq. (3)).

(3) $$\text{λ=}\frac{\text{g}{\text{T}}^{2}}{2\text{π}}\text{tanh}\left(\frac{2\text{π}\text{H}}{\text{λ}}\right)\text{where }\text{k=λ/π.}$$

**Fig. 4:**
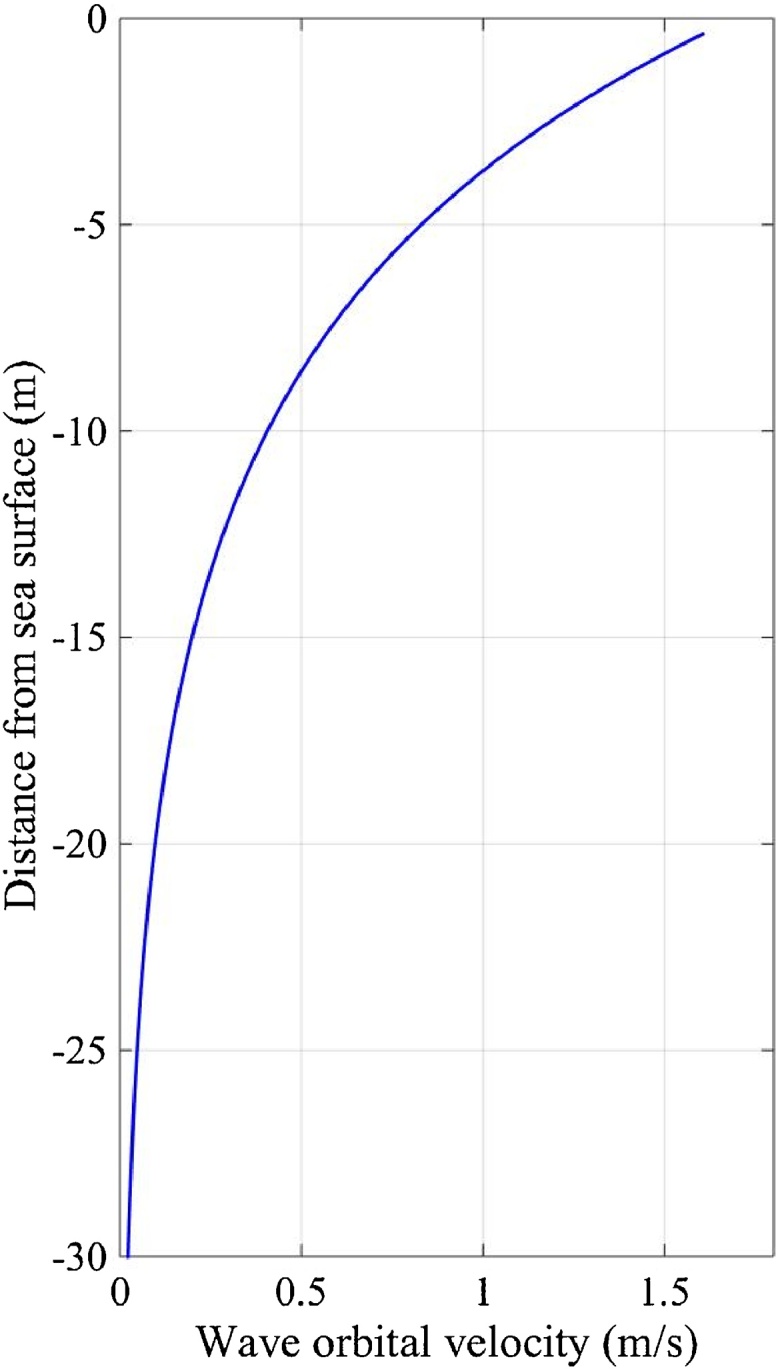
Predicted maximum-annual wave orbital velocity through water column using Eq. (1) (H = 3.99 m and t = 7.4 s) and data from Falmouth bay on 10/03/2013 between 19:46 and 21:16.

Where, λ is wavelength and g is gravity (9.81 m/s^2^). The characteristic maximum-annual wave dimensions in Falmouth bay occurred on 10/03/2013 between 19:46 and 21:16 (same data as maximum-annual current velocity, Fig. 3b) were 3.99 m significant height and 7.4 s mean period. The wavelength (λ) was predicted to be 44.0 m using Eq. (3) and an iterative process, (Sarpkaya and Isaacson, 1981). Therefore, the waves were considered deep water waves and $u_{deep}$ was calculated from Eq. (1). The vertical profile of wave orbital velocities through the water column (Fig. 4) showed high velocities near the sea surface (over 1.5 m/s) and exponentially decaying towards the sea bed. Vertical orbital velocity was equal but 180° out of phase to horizontal orbital velocity. The wave velocities near the sea surface are greater than the current velocity, thus were considered within the framework.

#### 3.3.3 Turbulent fluctuations

Turbulent fluctuations (u') are sporadic, high frequency changes in instantaneous velocity (u) around the mean velocity averaged over a defined period ($\bar{u})$, see Eq. (4) (Bouferrouk et al., 2016).

(4) $$\text{u=}\overset{-}{\text{u}}\text{+u'}$$

ADCP data has been used to calculate an example of turbulence fluctuations at WaveHub in Cornwall, UK. A 300 kHz 5-beam Workhorse Sentinel ADCP manufactured by Teledyne Technologies Incorporated, CA, USA, was deployed to measure with a 2 Hz sampling frequency, no on-board averaging and a bin height of 4 m with the first bin 6.18 m above the seabed. Data sets were periodically recorded for 34 min (4096 samples) with a 26 min pause. The 300 kHz ADCP has a stated accuracy of ±(0.05 m/s + 0.5% of measurement value), (Teledyne and Instruments, 2009). The low tide water depth was 37.5 m. The ADCP recorded between 30/08/2011 and 16/10/2011.

**Fig. 5:**
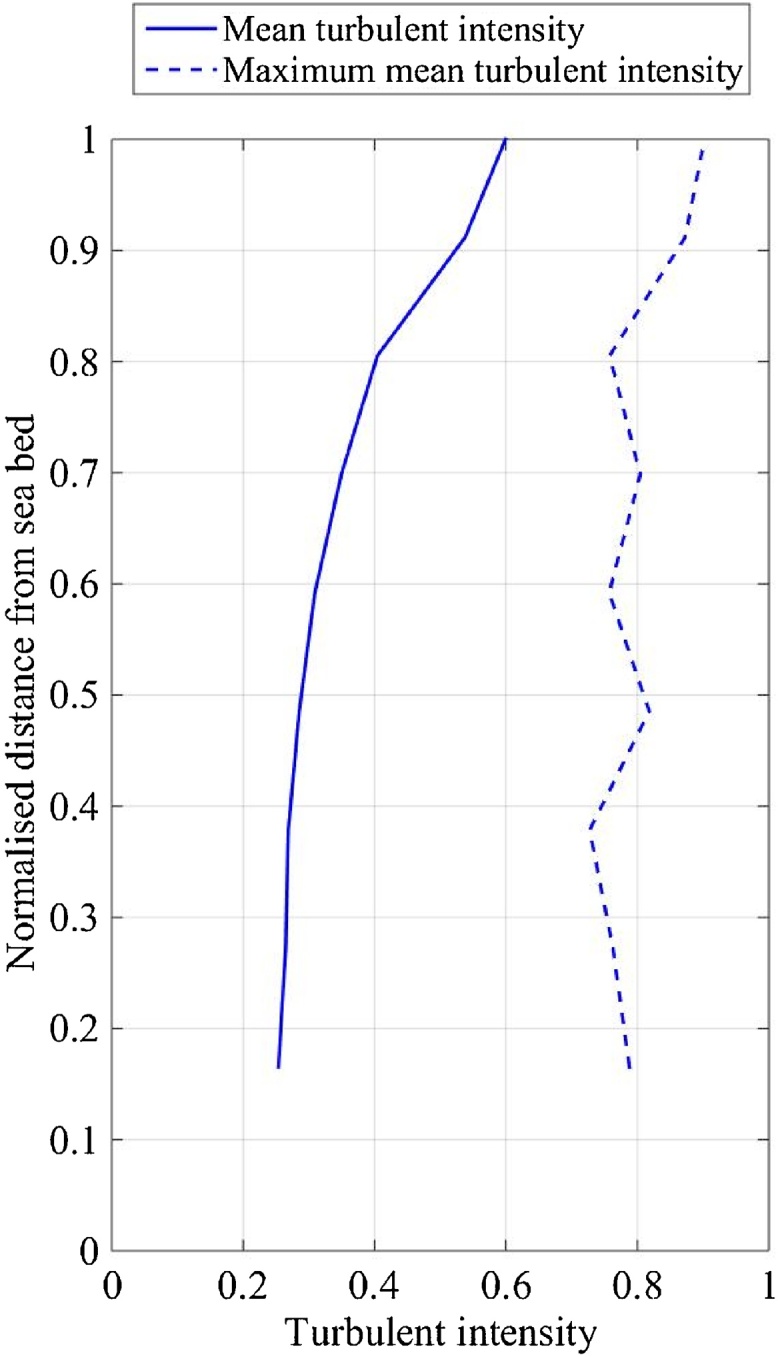
Normalised EVP of the 10 min-average of turbulent intensity measured at Wave Hub between 30/08/2011 and 16/10/2011. Low tide water depth was 37.5 m.

Turbulent fluctuations ($u_{RMS}^{'}$) can be normalised using the mean velocity ($\bar{u}$) to express the turbulence intensity (I), defined in Eq. (5). Normalisation was used to combine data from separate test sites by normalising turbulence and water depth (Fig. 5). The mean turbulence intensity varies from 0.6 near the sea surface to 0.25 near the seabed, however, the maximum mean turbulence intensity (taken as the maximum averaged data set, 4096 samples) is fairly constant through water column at a mean of 0.8, range of 0.72 to 0.9 (Fig. 5). The turbulent fluctuation magnitude is equivalent to 80% of the mean velocity, which emphasises the importance of considering turbulent fluctuations within the framework.

(5) $$\text{I=}{\text{u}}_{\text{RMS}}^{\text{'}}\text{/}\overset{-}{\text{u}}$$

#### 3.3.4 Dissolved oxygen concentration

**Fig. 6:**
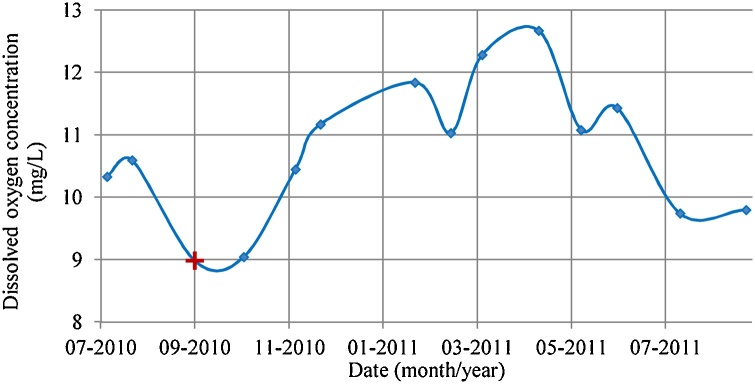
Annual variation (2010–2011) in near surface DO concentration in Falmouth bay, Cornwall, UK (public sector information licensed under the Open Government Licence v3.0). Cross marker (+) was used in the case study.

Oceanic DO concentrations collected from near surface samples in Falmouth Bay (Data obtained from public sector information licensed under the Open Government Licence v3.0 from 2010 to 2011) showed annual minimum DO concentration in Falmouth bay to be 8.98 mg/L (water temperature 15.9 °C). A year-round DO dataset (from 2010 to 2011), which spatially and temporally corresponds to the FaBTest current data, was used for the purpose of this study (Fig. 6).

### 3.4 Transfer functions

#### 3.4.1 Velocity profiles

Transfer functions were calculated from the results of an extensive series of hydrodynamic experiments performed in a current flume at the University of Exeter (Halswell et al., 2016). The experiments measured the relationship between external flow velocity and internal flow patterns with incremental percentages of biofouling coverage of four SBCC designs. The SBCC system shown to provide the most suitable rearing conditions from Halswell et al (2016), SBCC 1 at 90° angle of attack, was used in the present case study.

**Fig. 7:**
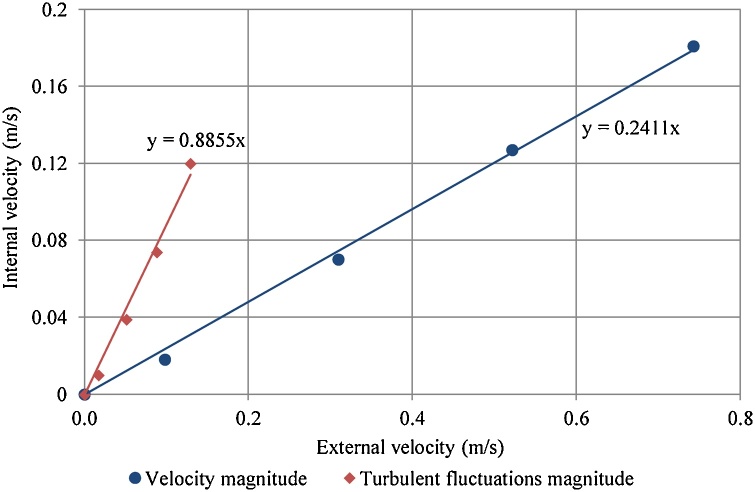
Relationships of velocity magnitude and turbulent fluctuations magnitude between external velocity and internal velocity from Halswell et al. (2016).

**Fig. 8:**
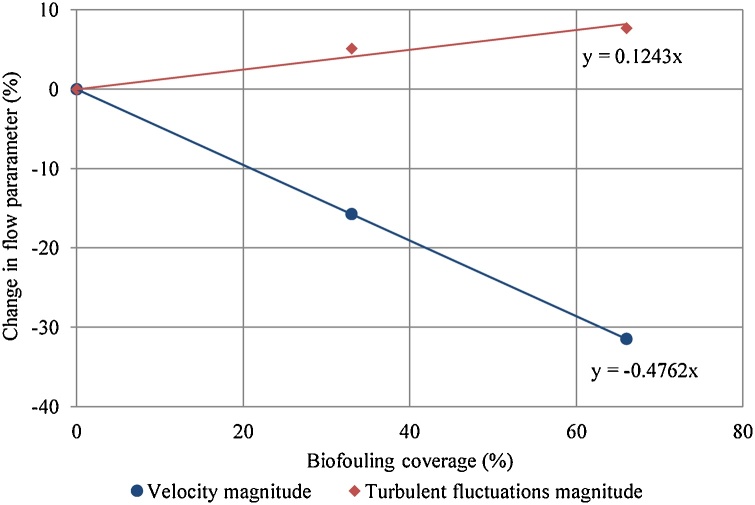
Relationships of velocity magnitude and turbulent fluctuations magnitude between internal flow velocity and biofouling coverage from Halswell et al. (2016).

EVP were extracted from characterisation experiments of model-bracket and end plates (Fig. 8, Fig. 9 of Halswell et al., 2016), and IVP from internal velocity measurement (Fig. 10, Fig. 11 of Halswell et al., 2016). A linear relationship (Eq. (6)) was assumed between EVP and IVP (Fig. 7), intercept axes at zero (c = 0) and regression analysis calculated the transfer coefficient (m). The limits of this linear assumption were discussed by Halswell et al. (2016) page 167.

(6) $${\text{u}}_{\text{in}}\text{=}{\text{m.u}}_{\text{ex}}\text{+c}$$

Where $u_{in}$ is internal velocity (m/s), $u_{ex}$ is external velocity (m/s), m is transfer coefficient and c is constant (0).

The relationship between internal and external velocity (Fig. 7) shows that internal turbulent fluctuation was not signifcantly affected by the SBCC; internal turbulent fluctatation was aproximately 89% of the external turbulent fluctation. However, the velocity magnitude was substantially reduced by the SBCC; internal velocity was aproximately 24% of the external velocity.

Transfer functions for biofouling were also required to predict how increased biofouling coverage affects the internal velocity over the deployment period, which accounts for temporal variations. The effect of 33% and 66% biofouling coverage was shown in Fig. 18 of Halswell et al., 2016. A linear relationship (Eq. (7)) was assumed between internal velocity and biofouling coverage (Fig. 8), intercept axes at zero (c = 0) and regression analysis calculated the constant (n).

(7) b=n.Bio+c

Where b is internal velocity variation (%), Bio is biofouling coverage (%), n is transfer coefficient and c is constant (0).

The effect of biofouling on internal flow velocity (Fig. 8) shows that increasing the biofouling coverage reduced the internal velocity magnitude (as expected); however, increasing the biofouling coverage increased the turbulent fluctuations.

The IVP was predicted from the EVP using Eq. (8). The first half of the equation predicts the mean current velocity (_cur_) and the second half predicts the turbulence velocity (_turb_); the combination defines the peak instantaneous velocity from Eq. (6). The transfer function coefficients are summaries in Table 1.

(8) $${\text{u}}_{\text{in}}\text{=}\left\{{\text{u}}_{\text{ex}}\text{.}{\text{m}}_{\text{cur}}\text{.}\left(\text{1+}{\text{b}}_{\text{cur}}\right)\right\}\text{+\{}{\text{u}}_{\text{ex}}\text{.}{\text{I.m}}_{\text{turb}}\text{.(1+}{\text{b}}_{\text{turb}}\text{)\}}$$

**Table 1:** Summary of transfer function coefficients.

Current		Turbulence	
$m_{cur}$	0.2411	$m_{turb}$	0.8855
$n_{cur}$	−0.4762	$n_{turb}$	0.1243
–	–	I	0.8

#### 3.4.2 Dissolved oxygen

A transfer function was used to convert the DO concentration limit for optimal growth (6.4 mg/l) into a velocity limit, for comparison to the IVP. The DO velocity limit ($u_{DO}$) was calculated based on the rate of DO supply and consumption using Eq. (9); assuming that DO is only supplied by replenished sea water as it was not possible to predict inputs from alternative biological sources. It also assumes the lobster has reached the maximum size for an SBCC container and has a fixed, maximum consumption rate. Therefore, this provided the worst case scenario for DO consumption rate.

(9) $${\text{u}}_{\text{DO}}\text{=}\frac{\text{L}}{\text{t}}$$

Where the length (L) of the multiple SBCC systems (SBCC containers continuously, i.e. no empty space between SBCC containers, moored along a line) was 97.6 m^2^ and the time constant of available DO (t) was calculated using Eq. (10):

(10) $$\text{t=}\frac{\text{V(}{\text{DO}}_{\text{sea}}-{\text{DO}}_{\text{lobster}}\text{)}}{\text{C}}$$

Where the annual minimum DO concentration in sea water (${DO}_{sea}$) was 8.98 mg/L (Fig. 6), the lowest acceptable DO concentration for optimal growth (${DO}_{lobster}$) was 6.4 mg/L (Drengstig and Bergheim, 2013) and the maximum DO consumption rate (C) of a 150 g lobster (the smallest lobster tested by Hamelo and the potential size of grown lobster in the rearing period) was 5 × 10^−5^ mg/s (Hamelo, 2006). Assuming the above, the time period to reduce the DO concentration within a container to below the lowest acceptable DO concentration for optimal growth (${DO}_{lobster}$) with no flow through the container is 15.9 h. Therefore, the DO velocity limit of 1.70 mm/s was calculated. The DO velocity limit is dependent on a number of parameters (DO level in the surrounding seawater [which is dependent on temperature, atmospheric pressure and salinity], size of lobster, size of SBCC array and other environmental factors [e.g. biofouling]). This limit is considered a constant in the present case study; however, its use should be recalculated for other applications, with appropriate transfer functions, DO consumption rates and DO concentrations.

### 3.5 Internal parameters

The EVP of current velocity, wave velocity and turbulent fluctuations were transformed into IVP using the transfer functions (Section 2.4).

#### 3.5.1 Current and wave velocity

**Fig. 9:**
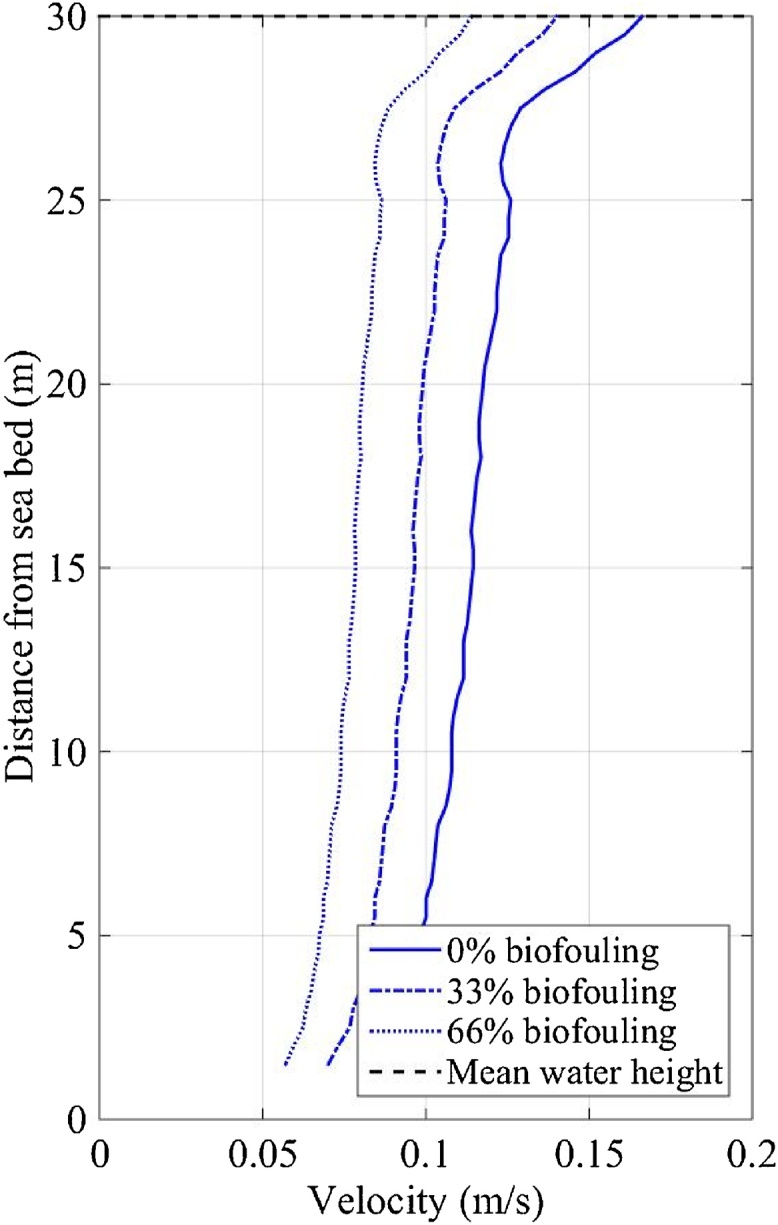
Maximum-annual IVP of the current and wave magnitude predicted from data collected on 10/03/2013 between 19:36 and 21:16 at SWMTF site.

The maximum-annual IVP of current and wave velocity (Fig. 9) predicted (using Eq. (8)) from the maximum-annual EVP measured on 10/03/2013 between 19:36 and 21:16 at SWMTF (Fig. 3b) followed the same trend through the water column as the maximum-annual EVP but was approximately a quarter of the magnitude; 33% and 66% biofouling coverage reduced the IVP (Fig. 9) by a further 15% and 30% respectively.

#### 3.5.2 Turbulent fluctuation

**Fig. 10:**
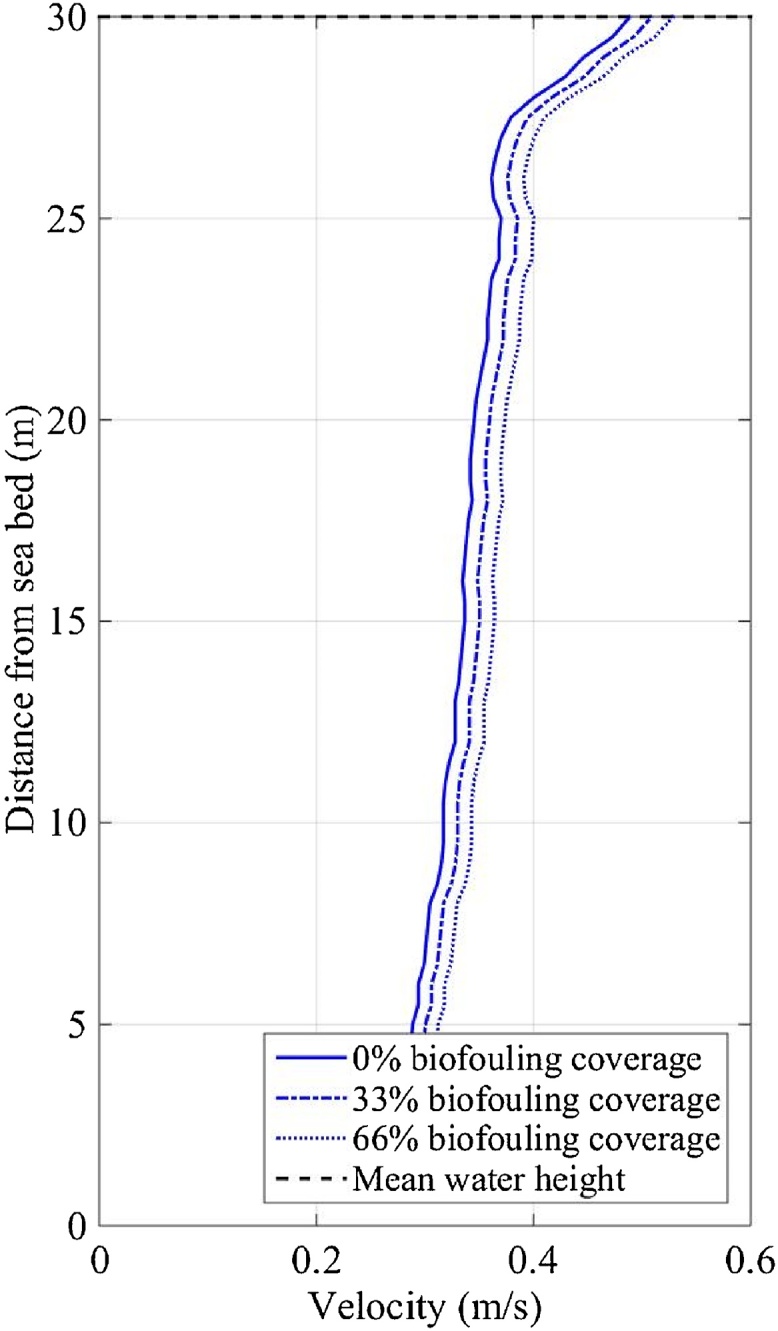
Maximum-annual IVP of the turbulence fluctuation magnitude predicted from data collected on 10/03/2013 between 19:36 and 21:16 at SWMTF site.

The maximum-annual IVP of turbulent fluctuations (Fig. 10) predicted (using Eq. (8)) from the maximum-annual EVP measured on 10/03/2013 between 19:36 and 21:16 at SWMTF (Fig. 3b), again, follows the same trend through the water column as the maximum-annual EVP; however, the IVP magnitude of turbulent fluctuation is approximately three times that of the current and wave IVP magnitude. Contrary to the current and wave velocity, the turbulent fluctuations increased with increasing biofouling coverage. Biofouling coverage of 33% and 66% increases the turbulent fluctuations by 4% and 8% respectively (Fig. 10).

## 4 Results

### 4.1 Rearing evaluation

#### 4.1.1 Dissolved oxygen

**Fig. 11:**
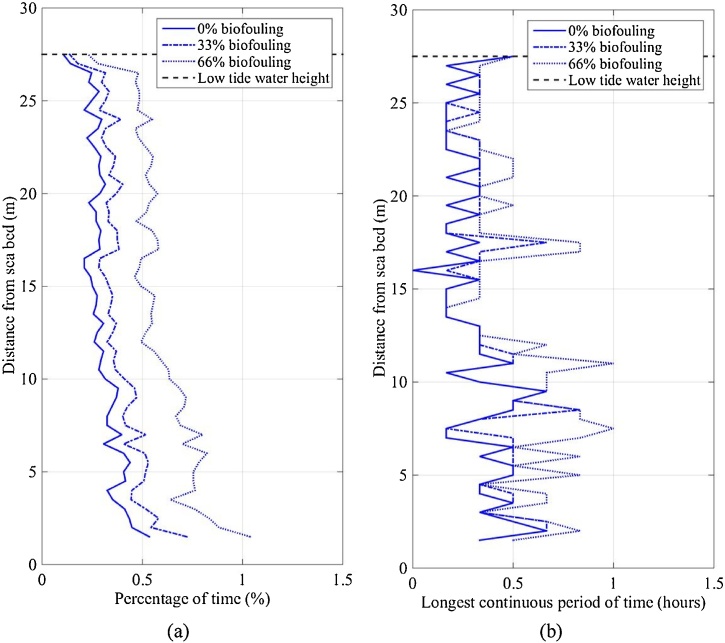
(a) Percentage of time and (b) longest continuous period of time that the IVP was less than the DO velocity limit (1.70 mm/s) at SWMTF between 16/09/2010 and 03/06/2011.

The DO velocity limit was 1.70 mm/s (Section 2.4.2) and only the mean current velocity was included when evaluating the effect of DO concentration on rearing success. The percentage of time that the IVP was less than the DO velocity limit was calculated from the measurements at SWMTF (nearly a year in duration). The percentage of time that the IVP was below the DO velocity limit was 0.25% at the sea surface and increased to 0.5% near the seabed (Fig. 11a). Furthermore, as the percentage of biofouling coverage increased, the percentage of time consequently increased to 0.5% at the sea surface and 1% near the sea bed. This indicates that positioning SBCC containers near the seabed is likely to provide less optimal DO conditions for rearing lobsters than the containers positioned near the sea surface.

On the other hand, it takes 15.9 h for a lobster to reduce the DO concentration below the acceptable DO concentration for optimal growth (Section 2.4.2). The percentage of time does not consider the time period between the occurrences of the IVP falling below the DO velocity limit. Thus the longest continuous period of time that the IVP was less than the DO velocity limit should be considered (Fig. 11b). At 0% biofouling coverage, the longest continuous period of time was approximately 0.25 h but increased slightly towards the seabed, to a maximum of 0.6 h. At 66% biofouling coverage, the longest continuous period of time peaked at 1 h, but the variation from sea surface to seabed was slightly greater. The longest continuous period of time at any percentage biofouling coverage did not exceed 1 h, as such, even though SBCC containers near the seabed provided less DO, growth should not have been affected when considering this rearing limitation. Thus DO supply should not affect the rearing success at any depth in this case study.

#### 4.1.2 Behaviour

**Fig. 12:**
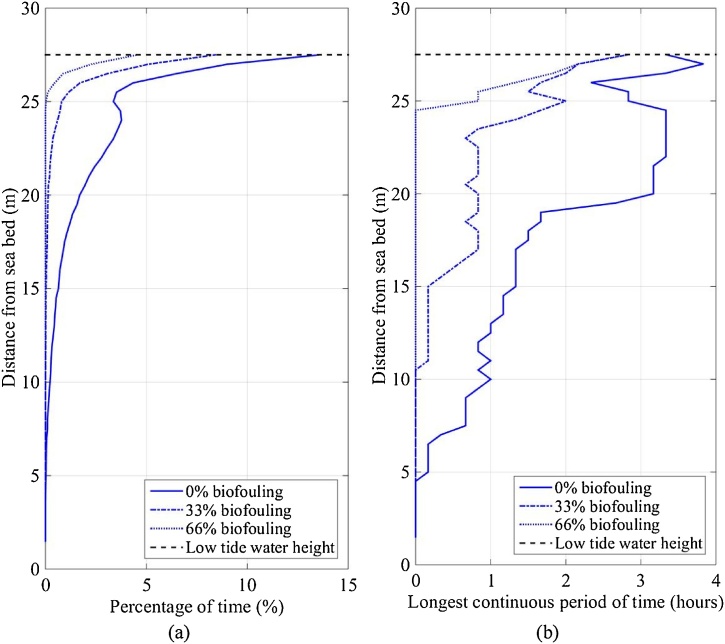
(a) Percentage of time and (b) longest continuous period of time that the IVP of current was greater than the foraging velocity limit (0.1 m/s) at SWMTF between 16/09/2010 and 03/06/2011.

The foraging velocity limit of 0.1 m/s was defined (Section 2.2.1 or Howard and Nunny, 1983). The percentage of time and longest continuous period of time that the IVP of current was greater than the foraging velocity limit (Fig. 12) was 12.5% and 3.5 h at the sea surface and decreased to 0% and 0 h near the seabed, respectively, with no biofouling. This indicated that the foraging behaviour of lobsters in SBCC systems was adversely affected at the sea surface and decreasing to no adverse effect near the seabed. Biofouling coverage decreased the IVP and as such the IVP spent more time (percentage and longest continuous period) below the foraging limit as biofouling coverage increased.

**Fig. 13:**
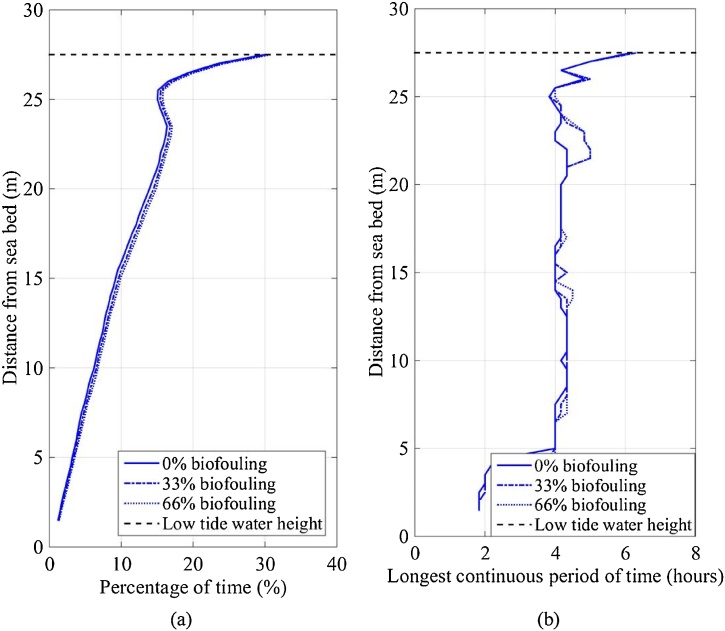
(a) Percentage of time and (b) longest continuous period of time that the IVP of current and turbulence was greater than the mobility velocity limit (0.27 m/s) at SWMTF between 16/09/2010 and 03/06/2011.

It was found that the IVP of current (i.e. excluding turbulence) never exceeded the mobility limit of 0.27 m/s. Indicating that the SBCC system was appropriate for use in Falmouth bay as the limit was never exceeded. However, results noticeably changed when turbulence was included (Fig. 13) as IVP increased by approximately 80% on top of the current velocity. Howard and Nunny did not measure turbulence fluctuations during the experiment, nor consider it during the analysis; thus no scientific literature exists relating to the effect of turbulence on mobility to the authors’ knowledge. Logic suggests that a lobster will have greater mobility when an IVP is uniform, regular and laminar (rather than varied, sporadic and turbulent) because the lobster can better predict the hydrodynamic forces that inhibit mobility; however, this requires scientific proof and quantification. The percentage of time and longest continuous period of time that the IVP of current and turbulence was greater than the mobility velocity limit (Fig. 13) was 30% and 6 h at the sea surface and decreased to 2% and 2 h near the seabed respectively. The combined IVP of current and turbulence has been included to highlight the need for research into the effect of turbulence on lobster behaviour and demonstrate the potential impact of turbulence on behaviour.

### 4.2 Evaluation framework

The evaluation framework has: predicted whether a geographical location (Falmouth bay) provided the required flow velocity for rearing success in the SBCC design, or vice versa, whether the SBCC design (i.e. the transfer function) was suitable for a geographical location; highlighted the flow requirement likely to have the greatest effect on rearing success; considered the temporal variation of the SBCC system by accounting for biofouling growth; and revealed the optimal vertical position of the aquaculture systems in water column, based on the parameters considered in the framework.

The Falmouth bay case study indicated that the IVP was too high because foraging activities were affected for noticeable periods of time (up to 15% near the sea surface); however, the internal DO concentration was never predicted to drop below the optimal DO concentrations. Thus lobster SBCC systems in Falmouth bay should be vertically located nearer to the seabed to decrease the percentage of time that the IVP is above the foraging velocity limit, based on the parameters included in the framework. However, this does not consider variations in vertical distributions in food availability, such as plankton where abundance is greater in the surface waters than at depth (Holligan et al., 1984) and thus could alter this suggestion. This highlights the importance of considering multiple rearing limitations to optimise positioning and thus rearing success.

## 5 Discussion

The authors acknowledge that the framework is a preliminary assessment and, as such, it requires further work and has limitations that will be discussed in this section. The framework used non-localised, (near) year-round data from which wave velocity and turbulence fluctuations were predicted. The use of non-localised data does provide some inconsistences; however, this does not affect or invalidate the framework as the aim of paper is to demonstrate the framework, not to provide generic results on rearing success. Furthermore, the rearing success parameters were derived from various literatures and were not tested in the case study area. To verify the framework, localised data sets and statistical observations of the successfulness of lobsters reared in SBCC systems are required. Field trials (Lobster Grower 2 – www.lobstergrower.co.uk) are currently underway in St. Austell bay, Cornwall, with approximately 35,000 European lobsters being deployed in SBCC systems between 2016 and 2019, which will feed in vital information required to validate the framework.

The framework focused on hydrodynamic parameters (current, wave and turbulence) alongside DO and behaviour as previous studies have quantified the effect of these parameters on growth and survival. However, other external parameters affecting rearing success in mariculture could be included, such as food availability, excretion waste removal, water temperature, salinity, concentration of heavy metals, motion severity, biological influences of biofouling, etc. The framework was developed with the flexibility to include additional parameters as scientific knowledge is gained.

The authors also acknowledge that turbulence was only modelled to affect mobility; however, it will also affect the settling of plankton inside the SBCC containers (Ross, 2006) and the disposing of sediment and excrement. Furthermore, the turbulent intensity here was depth averaged. Future study should enhance the inclusion of turbulence.

The velocity limits in this framework were fixed values; however, future model developments should consider spatial and temporal variations alongside variation dependent on other parameters. This paper used the minimum-annual DO concentration to calculate the DO velocity limit, which provided a conservative limit, but future models should explicitly compare the temporally varying DO velocity limit to a temporally varying IVP. A similar explicit comparison could be performed with the horizontal variation of DO concentration and additional parameters if the data was available. Furthermore future field trials should quantify the internal and external DO concentration differences considering presence of biofouling and other biological factors that could affect DO.

The framework presented here focuses on lobster SBCC systems; however, the framework is not exclusive and could be used for other species or systems in future. The velocity limits can be recalculated for other aquaculture species if the literature is available. Alternatively, transfer functions for alternate aquaculture systems could be derived from laboratory experiments similar to Halswell et al. (2016) or an EVP from another deployment location could be used.

Theoretically this framework could develop sufficient detail to assess the potential of multi-trophic aquaculture and/or colocation with other marine industries, both growing consideration for the mariculture sector. For example, if SBCC systems were co-located with offshore renewable energy devices that affect current, wave and turbulent velocities, then the effect on rearing success could be understood prior to investing in infrastructure.

A fully developed framework considering further parameters has the potential to be used by SBCC stakeholders for a number of advantages. First the framework can help reduce setup time and costs, by predicting the optimal position of SBCC systems based on environmental conditions. The framework could also increase growth rates and thus yield, by identifying optimal conditions for lobsters, because the current conditions are predefined by the SBCC design and external parameters, which have historically (prior to Halswell et al., 2016) been selected through speculative assumptions, rather than analytical predictions. The framework could also indicate appropriate maintenance intervals based on biofouling growth to sustain optimal conditions during temporal variations; biofouling provides a source of food (not captured in the presented framework) but also affects the DO supplied so the problem could be analytically optimised. Finally a fully developed framework has potential to model all the inputs and outputs of the SBCC system, thus increasing the accuracy of an environmental impact assessment.

## 6 Conclusions

The paper has presented a framework to evaluate the rearing success of European Lobsters in SBCC systems. The spatial and temporal variations of the external parameters (current, wave, turbulence, DO and behaviour) were transferred into internal parameters. The internal parameters were compared to DO and behavioural velocity limits to statistically quantify the rearing success. The results indicated that lobsters should be located near the seabed to increase foraging time whilst not affect DO availability. However, the framework is limited by the current level of knowledge regarding external parameters and it does not currently include food availability or consumption. The framework has been designed to allow new parameters and knowledge to be included in future versions, thus further work is required for the framework to reach full potential.

## Footnotes

1 The term IVP describes the flow velocity pattern inside the SBCC container that is experienced by the lobsters.

2 97.6 m was a constant, arbitrary number used based SBCC 1 of Halswell et al., 2016. Discussions with a mussel aquaculture farmer suggested that an initial full-scale deployment plan was a 200 m farm with a stack of SBCC containers every metre. SBCC 1 was 0.488 m diameter so the continuous length was 97.6 m (L = 200 x 0.488).
